# Cutaneous Bacteria of the Redback Salamander Prevent Morbidity Associated with a Lethal Disease

**DOI:** 10.1371/journal.pone.0010957

**Published:** 2010-06-04

**Authors:** Matthew H. Becker, Reid N. Harris

**Affiliations:** 1 Department of Biological Sciences, Virginia Tech, Blacksburg, Virginia, United States of America; 2 Department of Biology, James Madison University, Harrisonburg, Virginia, United States of America; University of Kansas, United States of America

## Abstract

Chytridiomycosis, caused by the fungal pathogen *Batrachochytrium dendrobatidis* (*Bd*), is an infectious disease that causes population declines of many amphibians. Cutaneous bacteria isolated from redback salamanders, *Plethodon cinereus*, and mountain yellow-legged frogs, *Rana muscosa*, inhibit the growth of *Bd in vitro*. In this study, the bacterial community present on the skin of *P. cinereus* individuals was investigated to determine if it provides protection to salamanders from the lethal and sub-lethal effects of chytridiomycosis. When the cutaneous bacterial community was reduced prior to *Bd* exposure, salamanders experienced a significantly greater decrease in body mass, which is a symptom of the disease, when compared to infected individuals with a normal bacterial community. In addition, a greater proportion of infected individuals with a reduced bacterial community experienced limb-lifting, a behavior seen only in infected individuals. Overall, these results demonstrate that the cutaneous bacterial community of *P. cinereus* provides protection to the salamander from *Bd* and that alteration of this community can change disease resistance. Therefore, symbiotic microbes associated with this species appear to be an important component of its innate skin defenses.

## Introduction

Infectious diseases of wildlife are emerging at an increasing rate and threaten global biodiversity [1]. The rapid emergence of these diseases may be a result of the alteration of community structures and relationships within ecosystems [1]–[3]. This hypothesis is based primarily on communities of macroorganisms, but alteration of the community structure of symbiotic microorganisms may also pose a risk for disease emergence [4]–[6]. One emerging infectious disease, chytridiomycosis, is a major factor causing drastic declines and extinctions of amphibian species in many parts of the world [7]. Chytridiomycosis is caused by the chytrid fungus *Batrachochytrium dendrobatidis* (*Bd*), which colonizes the skin of amphibian hosts [8]. There is no published evidence suggesting that the adaptive immune system (e.g. antibody production) is effective in controlling *Bd*. However for some amphibian species, innate skin defenses, including the microbial community of the skin and antimicrobial peptides secreted by the granular glands, can provide defense against the pathogen [9], [10]. In this study, we tested the hypothesis that alteration of amphibians' cutaneous microbial community can be associated with disease outcome.

Persistence with *Bd* varies among and within amphibian species [11]–[13] and has been associated with a species' assemblage of antimicrobial peptides and cutaneous microbial community [14]–[21]. *In vitro* studies and surveys have shown that bacteria isolated from amphibian skin produce strong anti-*Bd* metabolites and these metabolites are present on the skin in high enough concentrations to kill *Bd* zoospores and prevent disease [16]–[20]. In addition, field surveys of populations of the threatened frog *Rana muscosa* have shown that declining populations are characterized by having a relatively low proportion of individuals with anti-*Bd* skin bacteria. However, populations coexisting with the pathogen have significantly higher proportions of individuals with protective bacteria [21]. Bio-augmentation studies suggest that inoculating amphibians with anti-*Bd* bacteria prior to infection prevents morbidity and mortality by bacterial production of antifungal metabolites [16], [17]. The anti-*Bd* bacterial species *Lysobacter gummosus* isolated from the salamanders *Plethodon cinereus* and *Hemidactylium scutatum* produces the anti-*Bd* metabolite 2,4-diacetylphloroglucinol [19]. In addition, the bacterium *Janthinobacterium lividum* isolated from *P. cinereus, H. scutatum*, and *R. muscosa* secretes the anti-*Bd* compound violacein. This compound inhibits *Bd* growth at relatively low concentrations (minimum inhibitory concentration equals 1.8 µM) [20].

The specific aim of this study was to determine if the bacterial community on the skin of amphibians inhibits the growth of *Bd in vivo* by testing whether salamanders with reduced skin bacteria experience greater mortality and morbidity from *Bd* when compared to individuals with a normal cutaneous microbiota. The ability of cutaneous bacteria to inhibit the effects of *Bd* was evaluated by measuring aspects of salamander health, such as change in mass and survival, behavior, and the approximate quantity of *Bd* zoospores present on the amphibians' skin during the course of the experiment.

## Methods

### Study species

*Plethodon cinereus* is a terrestrial salamander with a geographic range spanning across most of the northeastern United States, southern Quebec, and the Maritime Provinces of Canada. This species is highly abundant within its range. Their moist, nutrient-rich skin helps support a diverse community of cutaneous bacteria [22], [23]. Bacterial isolates from the salamander inhibit the amphibian fungal pathogens *Mariannaea* sp. [23] and *Batrachochytrium dendrobatidis* [10]. There is no evidence that *P. cinereus* is affected by chytridiomycosis in nature, but this species can be infected by *Bd* [17]. Therefore, this species was a good candidate to determine if the cutaneous microbial community is responsible for the apparent resistance of *P. cinereus* to chytridiomycosis.

### Sampling and housing

Fifty-five adult salamanders were collected near Bother Knob in the George Washington National Forest in Rockingham County, Virginia. Cross contamination between samples was prevented by using instant hand sanitizer containing ethyl alcohol between each capture. The salamanders were brought into the laboratory within 24 hours where they were weighed and swabbed for the presence of *Bd*. Swabs (MW100 fine-tip; Medical Wire & Equipment, Corsham, Wiltshire, England) were drawn across the skin of the ventral side 10 times and each of the lateral sides 10 times. Swabs were frozen immediately at −80°C until further processing. Salamanders were individually rinsed in sterile artificial pond water [24] to remove transient bacteria from the skin before swabbing [23]. Salamanders were housed in individual 17×12×7 cm (L×W×H) sterile plastic containers lined with sterile filter paper soaked with sterile artificial pond water. Individuals were randomly placed at spots within incubators set at a temperature of 17°C and a 12-hour light cycle, both of which are representative of the species' natural conditions. On a weekly basis, salamanders were fed 10 fruit flies, which is an adequate feeding regime to maintain weight, after which their housing was cleaned and sterilized.

### Experimental design

Each individual was assigned a number and placed in treatments using a random numbers table generated from SAS statistical software (SAS Institute Inc., Cary, North Carolina). Four treatments (*n* = 55) were used to test the overall hypothesis that the microbial community is associated with disease outcome. The first treatment (*n* = 21) comprised individuals that had their cutaneous bacteria reduced prior to being exposed to *Bd* (Bac−Bd+). The second treatment (*n* = 8) comprised individuals that had their cutaneous bacteria reduced without being exposed to *Bd* (Bac−Bd−). This treatment controlled for any possible effects that the salamanders might have undergone as a result of the removal of their microbiota. The third treatment (*n* = 21) comprised individuals that retained their cutaneous microbiota in addition to being exposed to *Bd* (Bac+Bd+). Comparing this treatment to the Bac−Bd+ treatment allowed us to determine if the cutaneous microbiota inhibited the growth of *Bd*. The fourth treatment (*n* = 5) comprised individuals that retained their normal community of bacteria while not being exposed to *Bd* (Bac+Bd−). This treatment acted as a control for possible effects of the housing and handling of the salamanders throughout the experiment. Experimental treatments (Bd+) had a higher level of replication than control treatments (Bd−) because we wanted the highest statistical power for the comparison of most interest, i.e. does the presence of microbiota reduce the effects of chytridiomycosis?

### Bacteria removal

The treatments Bac−Bd+ and Bac−Bd− were exposed to both antibiotics and hydrogen peroxide to reduce the cutaneous microbiota on each individual. In a preliminary experiment, it was determined that three broad-spectrum antibiotics (9 µg/ml of sulfamethazine, 12 µg/ml of cephalexin monohydrate, and 3 µg/ml of trimethoprim) dissolved in artificial pond water in addition to an exposure of 3% hydrogen peroxide significantly decreased the mean number of morphologically distinct isolates (t-test, *t* = 4.012, d.f.  = 9, *P*<0.001). Hydrogen peroxide is also highly effective against bacteria [25]–[27]. Salamanders were placed in individual 50 ml Falcon tubes (Becton Dickinson, Franklin Lakes, New Jersey) with 15 ml of antibiotic solution for four hours. The Bac+Bd+ treatment and the Bac+Bd− treatment were exposed to artificial pond water for the same duration. After the antibiotic treatment, individuals were allowed a 24-hour period of rest before being exposed to a 3% percent hydrogen peroxide solution. Salamanders were put in a 25 ml bath of hydrogen peroxide for 30 seconds and then rinsed in artificial pond water. The Bac+Bd+ and Bac+Bd− treatment groups were exposed to artificial pond water for the same duration and then rinsed. After day 28, the Bac−Bd+ and Bac−Bd− treatment groups were exposed to an artificial pond water solution containing 200 µg/ml of streptomycin and 100 µg/ml of penicillin for seven days every other week. The antibiotic solution was used to moisten the filter paper lining the containers instead of artificial pond water as was used in the previous weeks. This was done to reduce bacterial growth on the salamanders. Penicillin and streptomycin do not inhibit *Bd*, as both antibiotics are used in cultures of *Bd* to prevent bacterial growth [28]. The treatments Bac+Bd+ and Bac+Bd− continued to be exposed to artificial pond water. Microbial richness was not sampled during the experiment to minimize stress to the salamanders that would be caused by additional swabbing. However, the use of identical antibiotic bath protocols in the preliminary and main experiments insured that microbial richness was reduced in the main experiment.

### *Batrachochytrium dendrobatidis* exposure

A strain of *Bd*, JEL 423, was obtained from J. E. Longcore (University of Maine). The strain was isolated from the frog *Phyllomedusa lemur* collected from El Copé, Panama on 17 December 2004 during a decline of amphibians due to *Bd*. This isolate was chosen because it was representative of a lethal strain of *Bd*. Culture maintenance and harvesting of zoospores followed methods published by Rollins-Smith et al. [29]. Each salamander in treatments Bac+Bd+ and Bac−Bd+ were exposed to approximately 18.7×10^6^ zoospores suspended in 5 ml of artificial pond water for 4 hours in 50 ml Falcon tubes. Tubes were turned every 30 minutes to ensure each individual was continually exposed to the pathogen. This day was considered day 1 of the experiment. Salamanders were then transferred to 8.5×7×6.5 cm (L×W×H) sterile plastic containers with the 5 ml *Bd* suspension for 24 hours. Bac+Bd− and Bac−Bd− individuals were treated in a similar fashion; however, they were placed in sterile artificial pond water.

### Masses, behavior, and mortality

Masses were taken initially when the salamanders were brought into the laboratory and at the conclusion of the experiment, day 55. Before masses were measured, salamanders were individually placed on sterile filter paper to remove excess moisture. Loss of mass is a symptom of chytridiomycosis and has been observed in *Bd*-infected *P. cinereus* individuals [17] as well as other amphibians [30], [31]. Mortality and behavioral changes (limb-lifting) were noted bi-weekly during the course of the experiment. Limb-lifting is defined as a salamander lifting one or more limbs off the substrate during a five minute observation period. During weekly checks, it was noted whether or not shed skin was present in the housing.

### *Batrachochytrium dendrobatidis* detection

Salamanders were swabbed for *Bd* four times during the experiment: initially and on days 14, 28, and 55. The same technique for swabbing was used as previously described. All swabs were frozen immediately at −80°C until further processing. DNA was extracted from the swabs using 50 ul of PrepMan Ultra (Applied Biosystems, Foster City, California), as described by Hyatt et al. [32]. The real-time Taqman PCR assay protocol, as described by Boyle et al. [33], with the addition of an internal control procedure to detect the presence of PCR inhibitors [32] was used to analyze the *Bd* swabs. Standards for the assay were obtained from D. Boyle (CSIRO, Geelong, Australia). Samples were amplified in triplicate with an Applied Biosystems 7500 real-time PCR system (Applied Biosystems, Foster City, California). The experiment was predetermined to last 55 days, which is representative of other *Bd* infection studies [30], [34]–[36], at which time all individuals were euthanized with methane tricainesulfonate.

### Statistical analyses

To determine whether removing bacteria affected body mass in uninfected individuals, we compared mean proportion of body mass lost in treatments Bac+Bd− and Bac−Bd− using a t-test. Because changes in body mass were statistically indistinguishable between these groups (t-test, *t* = 0.948, d.f.  = 9, *P* = 0.368), these control treatments were pooled for the remainder of analyses [37]. Proportion of body mass lost by the control group and the two experimental treatments, Bac+Bd+ and Bac−Bd+, was compared using a one-way analysis of variance (ANOVA). Fisher's least significant difference was used to test the *a priori* hypothesis that the Bac+Bd+ and Bac−Bd+ treatments differed from each other. After initial body mass measurements were made, four salamanders lost their tails and were removed from the mass analyses because it was unclear how these individuals' masses would change over time had they not lost their tails. Limb-lifting behavioral data and skin shedding data were analyzed with a Fisher's exact test to test the hypothesis that the proportion of individuals exhibiting limb-lifting or skin shedding depended on treatment.

## Results

### Body mass and mortality

**Figure 1:**
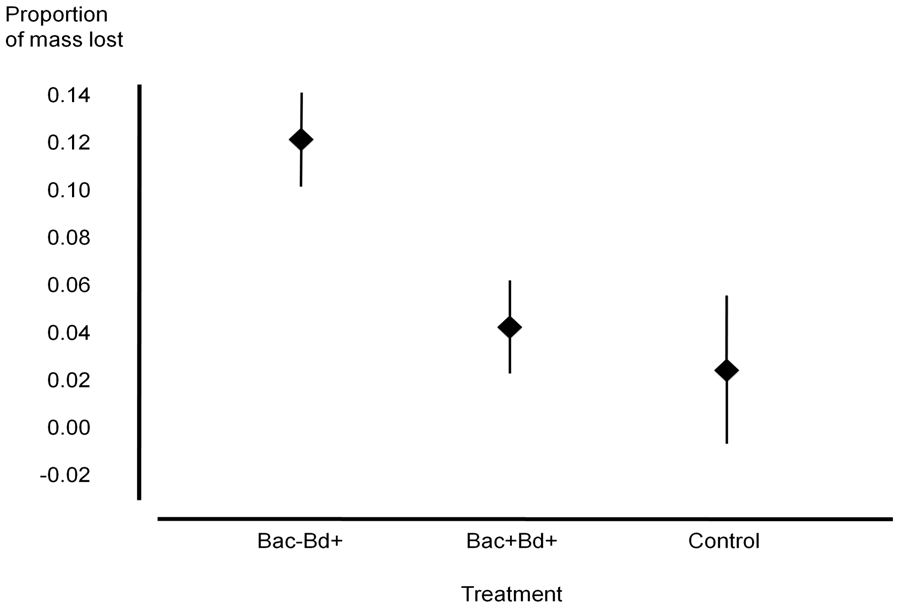
Proportion of body mass lost over a period of 55 days for treatments Bac−Bd+ (*n* = 20), Bac+Bd+ (*n* = 20), and controls (Bac−Bd− and Bac+Bd−, *n* = 11). Error bars represent ±1 standard error.

Bacterial removal did not affect change in body mass of uninfected individuals (t-test, *t* = 0.948, d.f.  = 9, *P* = 0.368). Over the course of the experiment, Bac−Bd+ salamanders lost three times as much mean body mass as Bac+Bd+ salamanders and four times as much as control individuals (ANOVA, *F*_(2,48)_  = 3.580, *P* = 0.036; Figure 1). The *Bd* strain JEL 423 did not cause mortality, as all salamanders were still living at the end of the experiment.

### Behavior and skin shedding

**Figure 2:**
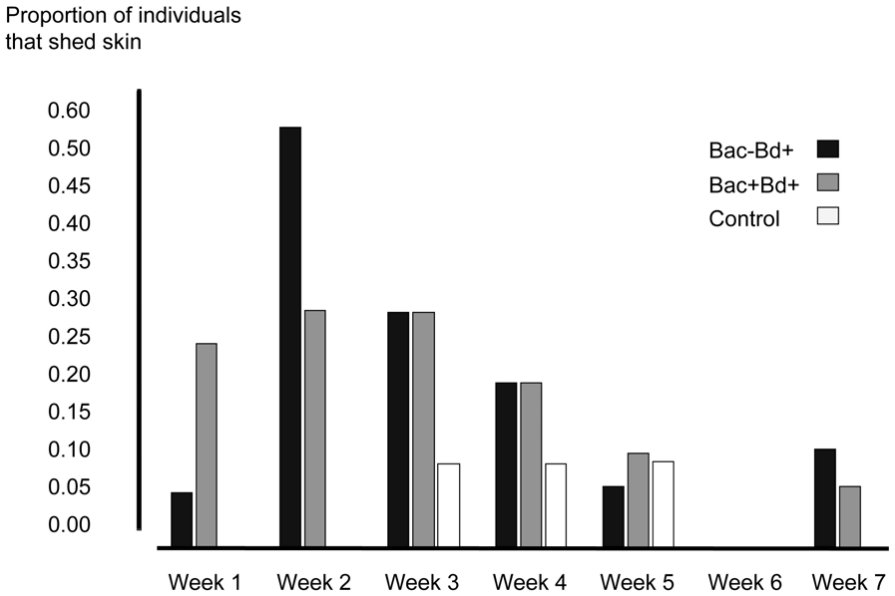
Proportion of individuals in treatments Bac+Bd+ (*n* = 21), Bac−Bd+ (*n* = 21), and control (*n* = 13) that shed skin each week.

**Figure 3:**
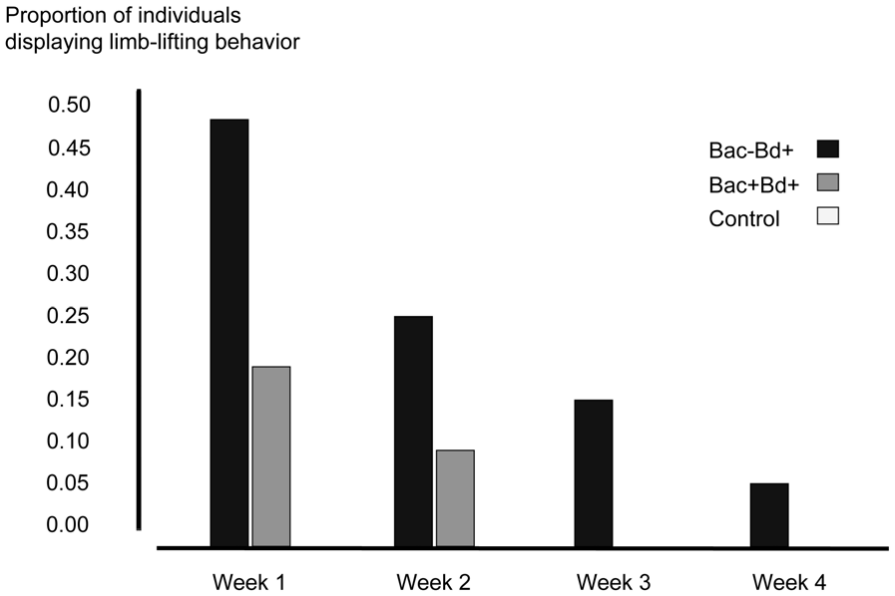
Proportion of individuals in treatments Bac+Bd+ (*n* = 21), Bac−Bd+ (*n* = 21), and control (*n* = 13) that lifted their limbs off substrate for at least five minutes each week.

Individuals exposed to *Bd* displayed signs of infection, such as excessive skin shedding and raising their limbs off the substrate. During the course of the experiment, a higher proportion of *Bd* exposed individuals shed skin (Fisher's exact test, d.f.  = 1, *P* = 0.035) and lifted limbs (Fisher's exact test, d.f.  = 1, *P*<0.001) than control salamanders. For individuals exposed to *Bd*, an equivalent proportion of individuals shed skin in treatments Bac−Bd+ and Bac+Bd+ (Fisher's exact test, d.f.  = 1, *P* = 0.253). For the two treatment groups exposed to *Bd*, skin shedding peaked during week two of the experiment and declined over the remainder of the study (Figure 2). Limb-lifting behavior peaked during week one, declined until week four (Figure 3), and was not observed again. During the course of the experiment, more individuals of the treatment Bac−Bd+ held their limbs off the substrate than Bac+Bd+ individuals (Fisher's exact test, d.f.  = 1, *P* = 0.031).

### Infection level

No zoospore DNA was detected on the skin before the salamanders were exposed to *Bd*. Throughout the experiment, mean numbers of zoospore equivalents as detected by real-time Taqman PCR were low for all individuals exposed to *Bd*. On day 14, individuals in the Bac+Bd+ and Bac−Bd+ treatments had an average of 0.39±0.79 (sd) and 0.491±0.56 (sd) zoospore equivalents, respectively, whereas individuals in the Bd− control treatment had no detectable zoospore equivalents. On days 28 and 55 of the experiment, all salamanders tested negative for *Bd*. Therefore, all salamanders were able to clear their infections by day 28, as determined by real-time Taqman PCR. The low levels of *Bd* detected at day 14 suggest that salamanders were able to clear infections rapidly even with a reduced skin microbiota.

## Discussion

This study demonstrates that the community of cutaneous bacteria on *P. cinereus* is an important factor preventing weight loss, a non-lethal symptom of chytridiomycosis, of infected individuals and thus is a component of the innate skin defenses. The lack of mortality and loss of infection seen in this experiment, even among individuals with their bacterial community reduced, is likely due to components of the innate defense system other than anti-*Bd* bacteria, such as antimicrobial peptide secretions and behavioral changes. Antimicrobial peptides have been found on a number of amphibian species and can inhibit *Bd in vitro* [9].

The low number of zoospore equivalents found on salamanders suggests that the infection subsided before the salamanders' skins were first assayed on day 14. In a comparable infection experiment with *P. cinereus*, similar infection intensities (weighted mean = 7.72 zoospore equivalents per infected individual) were found at day 14, suggesting that the pathogen is quickly cleared from the skin in this species of salamander [17]. In that study, histological examination of shed skins confirmed the presence of zoosporangia even though infection intensities were low. The observation that skin shedding and limb-lifting rates were greatest in the first two weeks and decreased thereafter provides additional evidence that an infection occurred early in the experiment and was quickly cleared. The difference in the change in body mass evident after 55 days suggests that individuals in the Bac−Bd+ treatment had a reduction in mass caused by infection prior to day 14, when the amount of *Bd* detected was low, and the reduction in mass was retained throughout the experiment. Individuals in the Bac+Bd+ treatment and control individuals maintained their body mass throughout the experiment. Interestingly, limb-lifting behavior has not been previously described for this species and its cause and function are unknown, but it might minimize transmission of *Bd* from moist soils in nature. The strict randomization included in the experimental design strongly suggests that significant differences in weight loss and limb-lifting behavior were due to treatment differences [38].

The beneficial effect provided by the bacterial community in this experiment may be attributed to a mutualism between the host and its microbes, which gain nutrients and substrate from the amphibian host. Similar mutualisms are seen in other host-symbiont models. For example, the beetle *Dendroctonus frontalis*, the wasp *Philanthus triangulum*, the leaf-cutter ant (Attini: Formicidae), and the lobster *Homarus americanus* all display mutualistic relationships with microorganisms. In all of these cases, the host is protected from a pathogenic fungus by a bacterial symbiont [39]–[42]. In these species, the bacterial symbiont produces antibiotic metabolites that inhibit the invading pathogen. For example, chemical analysis of a mutualistic actinomycetous bacterium isolated from its host, *D. frontalis*, has revealed that fungal infestation of the host is prevented by secretion of a polyene peroxide [42].

The protective effect provided by the bacterial community of *P. cinereus* may be a result of multiple ecological interactions with the invading zoospores. This beneficial community could prevent the colonization and inhibit growth of invading zoospores in at least three ways. First, the number of potential sites that zoospores can colonize is reduced by the presence of resident microbes. The ability of resident bacteria to prevent invading microorganisms by blocking adhesion sites has been demonstrated in past studies [43], [44]. Second, many bacteria have the ability to alter the microenvironment that they inhabit through secretory products [45], [46]. If the microenvironment of amphibian skin, such as its pH, is altered in a way that zoospores could not survive, this could prevent the colonization of *Bd*. Third, many bacterial species secrete antimicrobial compounds that directly kill other microorganisms. These antimicrobial compounds include products such as enzymes, bacteriocins, fatty acids, and hydrogen peroxide [47], [48]. Bacteria isolated from amphibians secrete secondary metabolites that inhibit the growth of *Bd in vitro* [19], [20]. For example, the bacterial species *Janthinobacterium lividum* isolated from the amphibian species *P. cinereus, Hemidactylium scutatum*, and *R. muscosa* secretes violacein and indole-3-carboxaldehyde. These compounds strongly inhibit the growth of *Bd* at low concentrations [20].

As suggested by this study, skin bacteria are not the only factor preventing chytridiomycosis. There are multiple aspects of an amphibian's innate defenses, which include sloughing of the skin and glandular secretions. In this study, *Bd* affected the rate of skin shedding. Sloughing of skin is a symptom of chytridiomycosis [49]. However, in the case of *P. cinereus*, this response may function as a way for the salamander to reduce the zoospore load and prevent severe infection. *Bd* resides entirely in the outer cell layers of the epidermis [50]. When *Bd* colonizes amphibian skin, it is hypothesized that a germ tube grows into the lower cell layers of the epidermis through which its intracellular contents are inserted into an epidermal cell [51]. The chytrid develops while normal renewal of skin layers occurs such that when zoosporangia are mature, they are at the outermost layer of amphibian skin [50]. Accelerating the rate at which skin is replaced could result in shedding of *Bd* before it has matured, thereby preventing the release of zoospores on the host. Accelerated shedding of the skin may be an effective response of amphibians in reducing the pathogen load on the skin. However, no difference in the proportion of individuals that shed their skins between Bac−Bd+ and Bac+Bd+ treatments was detected, meaning that skin shedding cannot explain the difference in weight loss observed between these treatments.

This study provides evidence that the cutaneous bacterial community is an important component of the innate skin defenses of *P. cinereus* that prevented weight loss associated with chytridiomycosis. Other defensive mechanisms, such as antimicrobial peptides, behaviors, and skin shedding rates have been shown to be important or are likely to be important in patterns of species-specific survival in response to chytridiomycosis. However, these mechanisms are not amenable to manipulation as a management tool in conservation. Manipulation of anti-*Bd* bacteria is a promising method that has the potential to allow re-introductions of amphibians from survival assurance (captive breeding) colonies into nature as well as to slow successful dispersal of chytridiomycosis in nature. In a laboratory experiment, bio-augmentation of the skins of mountain yellow-legged frogs, *R. muscosa*, with anti-*Bd* bacteria prevented morbidity and mortality due to *Bd* seen in individuals not treated with anti-*Bd* bacteria [16]. Taken together, the “bacteria removal” approach used in this study and the “bacterial addition” approach employed with *R. muscosa* provide evidence that alterations of microbial community structure can either enhance or decrease amphibian health. Further research is needed to determine the efficacy of bacterial manipulations as a means of preventing further declines of amphibian species due to chytridiomycosis.
